# A new paradigm for operative dentistry

**DOI:** 10.4103/0972-0707.43411

**Published:** 2008

**Authors:** Graham J Mount

**Affiliations:** Visiting Research Fellow, Faculty of Dentistry, The University of Adelaide, South Australia

**Keywords:** Operative dentistry, cariology, caries, dental history, paradigm

## Abstract

It is over 100 years since G V black gathered together most of the knowledge then current on the caries process and set clear parameters for the discipline of operative dentistry. His four-volume treatise set standards that were relevant for the times and, in fact, were so well described that they remained dominant in this discipline until quite recently. However, over the last 50 years there has been great progress in scientific method and in knowledge of the common diseases of the oral environment, including the caries process, so maybe it is time for change. The term “paradigm” describes a philosophy of science, a generally accepted model of how ideas relate to one another, forming a conceptual framework within which scientific research is carried out. Black defined the paradigm within which further research was to be conducted during the following years and the profession accepted his lead. However, it is not expected that the parameters of a profession should remain unchanged over a substantial period so it is suggested that the dental profession should, at this time, recognize a new paradigm. Improvements in scientific method have led to a better understanding of the oral environment, resulting in extensive changes for this profession. It is suggested that the standards set by Black should be now consigned to history and an entirely new paradigm adopted. First, the profession must recognize that dental caries is a bacterial disease and its primary efforts should be directed towards identification and elimination of the disease prior to initiating repair of the damage that it has caused. Preservation of natural tooth structure is then the next responsibility. There should be maximum use made of preventive strategies, including remineralization, followed by minimal intervention cavity designs and the use of bioactive restorative materials to restore the lesions. The profession should be prepared to move on.

## INTRODUCTION

The term “paradigm” describes a philosophy of science, a generally accepted model of how ideas relate to one another, forming a conceptual framework within which scientific research is carried out. At the beginning of the twentieth century GV Black[1] set out a paradigm for operative dentistry in his treatise on this subject published in 1908. In fact, he covered more than simple operative dentistry and extended the paradigm to cover the general clinical practice of dentistry including the study of subjects now known under specialist titles such as periodontology, endodontology and dental materials. He covered these fields in considerable depth delving back into history to explain the stages of development of the science as it stood at the turn of that century.

The problems of dental caries and periodontal disease go far back into history but Black entered the profession at a time when these diseases were endemic in the European and related communities. There had been very little by way of scientific literature published over the recent centuries but there was some discussion taking place on the causes, and therefore cures, available. Caries was regarded as “gangrene” of the tooth structure and it was not clear as to whether it began on the outside or the inside of the tooth crown. The roles of bacteria and food stuffs were hotly debated and the materials for restoration of lesions were relatively primitive. Tooth extraction was the most reliable method of treatment but the construction of dentures, both full and partial, was still in its infancy. There was a serious need to draw together all known facts related to dental disease and propose “a conceptual framework within which (further)scientific research (could be) carried out”.

## THE GV BLACK PARADIGM

The paradigm developed by GV Black was encapsulated within the first edition of his four-volume textbook, “Operative Dentistry”, published in 1908. It was just 40 years since Tomes,[2] using the very earliest version of a microscope, defined the histology of tooth structure and even less time since WD Miller,[3] working with Koch, first identified lactobacillus as an organism responsible for the generation of acid in the oral environment. Black settled a vigorous argument of the day by stating clearly that caries always begins on the outside of the tooth — never from within. He noted that “it consists of dissolution of calcium salts by lactic acid followed by decomposition of the gelatinous body of the organic matrix”. He went on to reprimand the profession stating clearly that “the complete divorcement of dental practice from studies of the pathology of dental caries, which has existed in the past, is an anomaly in science that should not continue”.[4] Within his textbook Black set the scene for a level of understanding that had been previously unknown to this profession. Drawing on the knowledge of the time he identified the primary causes of the disease of caries and he charted its progress through both enamel and dentine. Microscopy, microbiology and chemistry were in their infancy and Black used them to their full extent to demonstrate that bacteria were related to both caries and disease of the gums and surrounding soft tissue.

He rationalized the production and clinical application of amalgam for the restoration of lesions, set standards for dental cements and identified the three areas on the tooth surface where caries was most likely to begin — occlusal fissures, proximal contacts and cervical areas. Based upon this he developed a classification of caries lesions, with the numerical sequence dependent upon the frequency with which the lesions occur. Thus the Class I lesion represented the occlusal fissure, the most common site, and the Class V was the cervical lesion, the least common, and this classification is still in general use.

In a farsighted statement Black postulated that “the causes of immunity and susceptibility to dental caries would necessarily be found in conditions of the general system influencing the qualities of the mixed fluids of the mouth by which the teeth are surrounded”.[5] In other words he recognized that caries fulfils the dictionary definition of a “disease”, that is “a condition that results in medically significant symptoms in a human” and he offered a limited treatment range which was dictated largely by a lack of depth in the current understanding of the cause of the disease. He was limited to a mechanistic approach wherein the lesion was simply debrided and restored, without the ability to address the disease at a more fundamental level. As the disease would probably remain present and active, the cycle of debridement and restoration was likely to be repeated regardless of the quality of the restorative work. With “recurrence” of the disease further natural tooth structure would be lost and the problems of restoration would become more complex. This cycle ensured that dentists were kept busy to the extent that, even today, up to 75 per cent of a general practitioner's time is devoted to “replacement dentistry”. In other words, currently, the profession is “not winning the war”.

Shortly before his death in 1917, Black appeared to be on the brink of further discoveries inasmuch as, in conjunction with Robert Blackwell,[6] he wrote articles about “mottled enamel”. Fifteen years later both McKay[7] and Smith[8] discussed the relationship of the fluoride ion with mottled enamel and the connection with a reduced caries rate. It was a further 20 years before the profession developed an understanding of this relationship to the extent that it was able to persuade the public that there were real advantages in the incorporation of the fluoride ion into tooth structure. Thus, Black had led the way again and research continued. The profession at large accepted Black's paradigm and it formed the foundation for virtually all teaching of the general practice of dentistry for the remainder of the twentieth century. By the middle of the 1950s operative dentistry was the primary discipline of the profession and the literature of the time demonstrates the deep interest that had been generated. Obviously a profession cannot stand still. There will be constant clinical observation and laboratory research as well as developments in laboratory techniques, materials and equipment that will expand knowledge and stimulate further scientific investigations.

## IMPROVEMENTS IN TECHNOLOGY

In the first half of the last century there was fierce discussion as to the cause of caries with diametrically opposed camps. One group insisted that local environmental factors were primary and another group suggested that faulty diet and nutrition were the cause.[9,10] The only effective means of controlling the disease appeared to be surgical removal of the demineralized portion of an affected tooth along with any surrounding tooth structure that was considered to be at risk, that is “extension for prevention”. Dentists were known as “dental surgeons” because their main duty appeared to be surgical removal of tooth structure and there was little time devoted to teaching patients to understand the value of the preventive measures that were beginning to be understood.

By the second half of the century the knowledge and understanding of preventive measures had reached a reasonably sophisticated level and the profession was prepared to devote time to patient education. In many countries governments had been persuaded to incorporate fluoride into public water supplies and fluoride was being added to toothpaste.[11] This alone reduced the problems arising from the disease to a more manageable level[12] to the extent that the public at large began to understand that it was not inevitable that a person should lose all their teeth before the age of 40 years. From the 1940s to the 1970s there was considerable research carried out into restorative dental materials including clinical handling of amalgam,[13,14] perfection of casting techniques for gold alloys,[15] increased fracture resistance in dental ceramics[16] and improvements in impression materials.[17] Parallel with these developments came the introduction of high speed and ultra high speed rotary cutting instruments[18,19] as well as more effective local anaesthetics.[20] In 1955, Buonocore[21] showed that it was possible to gain mechanical adhesion between enamel and a polymethyl-methacrylate resin restorative material, thus overcoming microleakage which had always appeared to be one of the main problems of restorative dental materials. By the mid 1960s this had become a resin composite restorative material and there was considerable optimism that it would become the universal restorative material. However, up to the present time, they have not shown long-term adhesion to dentine, the problems of shrinkage during setting remain and the wear factor is still a debatable issue. Further research is required before their longevity is proven to be satisfactory. Nevertheless, adhesion to enamel has proven to be a major breakthrough along with the introduction of a level of aesthetics that had not previously been obtainable. In the last 40 years these materials have been thoroughly researched and reviews are available in a number of textbooks.[22,23] Ten years later, in the 1970s, there was a further adhesive material, the glass-ionomers, introduced to the profession.[24,25] These are also reasonably aesthetic and have been shown to adhere to both dentine and enamel through an ion exchange mechanism. They are waterbased and also bioactive in as much as they release calcium or strontium ions, as well as phosphate and fluoride ions, into surrounding tooth structure and these can assist in remineralization of a caries lesion.[26]

The changing trends in restorative materials were thoughtfully reviewed by Phillips[27] in 1989 and it became apparent that there had been a revolution in the treatment and understanding of the caries lesion over the last three decades. Both the profession and the public appreciated these improvements. The profession was no longer overwhelmed by rampant disease and there had been modification of the public attitude to the delivery of treatment. The fluoride ion had become a universal tool for stabilization of tooth structure for all age groups and remineralization techniques were becoming understood. Modifications to the oral flora became possible and the glass-ionomers had been shown to be bioactive. Aesthetic restorative materials and techniques became dominant and adhesion of the restoration to tooth structure became most important.

However, even now some of Black's teaching prevails, particularly in relation to cavity design. The Black cavity was designed for restoration with amalgam or cast gold and was based upon “extension for prevention” in the presence of continuing disease. At this time these designs are out of date and they can rightly be regarded as unduly destructive of natural tooth structure and should be abandoned or at least modified to accommodate the above factors. Also it seems that, in spite of this progress, there has been a decline in concentration on operative dentistry in parallel with a considerable increase in interest in other subjects. Each of the disciplines in turn has become dominant in dental teaching and thinking to the extent that the discipline of operative dentistry has fallen into the background. Back in the 1970s the stage was first occupied with the second most common disease- periodontal disease. This enjoyed a period of great interest followed by endodontics and then, logically, occlusion. Subsequently, crown and bridgework, followed by ceramics, became the principal interests and now it is dental implants.

These digressions are logical but it seems that they have been at the expense of operative dentistry, and this after all, is the core discipline if teeth are to be maintained in their natural form. There is a slowly growing recognition of the importance of retention of natural tooth structure but there seems to be some reluctance to abandon the principals originally laid down by GV Black. It is suggested that, at this time, cariology, followed by operative dentistry, should be the principal interest in both teaching institutions and clinical practice because if the disease of caries can be controlled then the parallel disciplines will be of less significance. After all, many of the common dental problems are, at least in part, iatrogenic in origin.

## THE SIGNIFICANT RESEARCH IN CARIOLOGY

It is essential that the profession understand how the disease is initiated in the oral environment and how it progresses. As early as 1967 Massler[28] drew attention to the changing concepts in the treatment of caries. Work by Kidd and Fejerskov,[29] Featherstone,[30] ten Cate[31] and many others, from the early 1970s onwards, helped to elucidate this. By 1980 Fusayama and his team was prepared to summarize their concepts on major modifications to cavity designs and the use of a total etch technique using resin composite as the restorative material.[32] Subsequently, it became apparent that the caries process could be arrested and in fact reversed.[33] Brännström[34] carried out pioneering work on the dental pulp and showed that its powers of recovery were greater than previously thought. Science then showed that the fluoride ion is an essential component in all age groups for the control of the disease. In recent times Reynolds[35] has developed a technique that will ensure remineralization of enamel in depth to the extent that it can eliminate the white spot lesion completely. In 2006, Ngo[26] published a paper showing that the glass-ionomers are bioactive and will assist in remineralizing dentine on the floor of a caries lesion.

The combination of this research opens the way to modifications in the approach of the profession to the problems still presented by the disease of caries. It must be noted that these investigations began over 30 years ago and the practicing profession has still not completely accepted the advantages that should have arisen from this accumulation of knowledge. It is suggested that the time has arrived for operative dentistry to regain its rightful place at the centre of both teaching and practice.

## THE NEW PARADIGM

The above review suggests that the magnitude of the changes in the science, understanding and clinical application of operative dentistry have already brought about a paradigm shift in the philosophy of this profession, even though it has not really been acknowledged. The development of the caries lesion is now recognized as an imbalance in the demineralization remineralization cycle, which is a natural phenomenon under the biofilm that is attached to the tooth surface. There is no doubt it is desirable that the early changes that lead to the caries process, prior to the formation of the “white spot lesion”, be recognized, with the assumption that the lesion can be avoided, or at least reversed, and cavitation of the lesion prevented. It has already been shown that, up to the point of surface cavitation, a white spot lesion can be healed. The profession, therefore, must be taught to assess the stability of the entire oral environment, note pathological changes and deal with these to bring about healing rather than simply reach for the handpiece and undertake the surgical repair of a lesion that still has the potential to be repaired, at least in part.

It has to be accepted, of course, that a lesion may progress beyond remineralization before the patient presents for consultation and therefore techniques for surgical repair cannot be dismissed. However, simply repairing the surface damage that has already occurred will not eliminate the disease. Surgical repair alone is not sufficient. Investigations into the cause and extent of the disease in the individual patient's oral environment are essential corollaries to treatment so it is essential to begin with elimination of the disease. Then, if surgical interference is required, it should be recognized that only that part of the tooth surface that is irreversibly broken down will need to be replaced because surrounding and underlying demineralized tooth structure can still be remineralized and healed and therefore need not be removed.[36] This means that the GV Black mantra of “extension for prevention” should be replaced with the concept of “prevention of extension”.

## FURTHER RESEARCH REQUIRED

Obviously, as scientific techniques evolve and knowledge progresses, any solution to a problem will pose as many questions as it does answers. There is almost certainly no end to the problems and it is just as likely that, before the end of this century, it will be suggested that the profession accept yet another paradigm as the basis for further research.

In the meantime there are a number of areas that require continuing research and some of these can be clearly defined. For example, following work by such investigators as Thylstrup and Fejerskov[37] the initiation of the demineralization /remineralization cycle is reasonably clearly defined. What is not yet fully understood are methods of reversing the cycle and reliably bringing about healing and remineralization and the time schedule involved. The potential for healing a caries lesion requires careful investigation and the proper mechanism must be defined.

### Remineralization with casein phosphopeptide-amorphous calcium phosphate

Calcium phosphate combinations (CPP-ACP)[35] has recently been proposed but requires further investigation particularly in relation to both time and method of application for best effect. Prescription routines for topical fluorides for resistance to demineralization, chlorhexidine for modification of bacterial flora and CPP-ACP for remineralization are currently essentially intuitive and do not take into account the varying levels of pathology in the oral environment of each patient.

The microbiology of the oral environment needs to be defined and reliable methods for identification of various strains of micro-organisms developed. There is a vast array of micro-organisms present in the oral environment at any given time and the interrelationship between the various strains is, as yet, poorly understood. The profession needs to be able to identify those bacteria related to pathology using simple, rapid and reliable in-surgery techniques so that the balance can then be reliably restored.

Techniques for the identification of the presence of the disease must be improved. At present there is still too much reliance on the early signs of demineralization and, in fact, some operators still rely on surface cavitation before acknowledging the presence of the disease. Even if the “white spot lesion” can be reversed, it is more desirable to be able to note changes in the environment when they first occur. The profession depends too much on radiographs to reveal lesions, particularly interproximally, and these are unreliable in the early stages of demineralization.

New techniques are under investigation and there is a need for refinement.[38]

It has been shown by Mertz-Fairhurst *et al.*[39] that complete isolation of the caries lesion is sufficient to arrest further progress over the long term. It has subsequently been shown that sealing a lesion with a bioactive restorative material, such as glass-ionomer, will enhance the potential for remineralization of underlying isolated demineralized dentine.[26] Research is required to define the extent of remaining remineralizable dentine on the floor of a cavity. Is it possible to clinically demonstrate the difference between “infected” and “affected” dentine as defined by Massler[40] Allied to this is the potential for actually rebuilding tooth structure through the application of stem cell research.[41]

There must be thorough investigations into the saliva flow and methods of countering a reduction in flow. This subject has been neglected but has now become very relevant particularly in the presence of the multi-pharmacology practised by the medical profession. Many drugs, alone or in combination, both therapeutic and recreational, are anti-sialogogues and therefore pose serious problems, particularly for both youth and the ageing and medically compromised patient.

All the direct restorative materials need further research particularly in relation to longevity in the oral environment. Gold and ceramics have long been regarded as the materials of choice because, properly constructed and in a mouth free of disease, they last many years. In recent years, largely based upon the euphoria of the aesthetic direct restoratives, the problems of longevity seem to have faded into the background. Obviously, replacement of any restoration will lead to further loss of natural tooth structure and is therefore to be avoided for as long as possible. Amalgam has been accused of bringing about bodily contamination through mercury and this controversy needs to be settled. Longevity of amalgam deserves further investigation because records are now available that demonstrate that, in the absence of disease, a restoration can last far longer than the published average of 12-15 years[42] with no apparent adverse effects. Resin composite requires further investigation in several aspects and efforts should be directed at in vivo investigations rather than in vitro if the problems of longevity are to be overcome. It is essential to eliminate setting shrinkage as well as the limited depth of cure, and the relatively low degree of conversion of the resin through light activation must be improved. Problems posed by the lack of adhesion to dentine can be overcome through lamination with glass-ionomers but enhanced wear resistance is required to ensure greater longevity. The significance and implications arising from the release of ions from the resin as it wears away need to be defined.

Glass-ionomer requires continuing research particularly in relation to its ability to enhance remineralization of demineralized tooth structure. The whole concept of the ion exchange between tooth structure and the cement needs to be investigated and, if possible, improved. The ion exchange adhesion to both enamel and dentine is the best method of adhesion available and is limited only by the physical properties of the glass-ionomer. Therefore these properties need to be enhanced, particularly increased resistance to fracture. Maintenance of the water balance in the newly placed restoration is important but the newer materials already show enhanced stability. The significance and implications from release of ions from the worn restoration need to be defined. The importance and longevity of the lamination technique with resin composite needs further investigation because this is the best way to provide union between resin composite and dentin.

If “extension for prevention” is now out of date just how much demineralized tooth structure can be reliably saved and healed? Several modifications to the cavity designs suggested by Black have already been clinically tested but so far there is no consensus on what is acceptable.[43] Of course, elimination of the disease is the essential prerequisite for success with any cavity design because obviously restoration of tooth structure will not cure the disease. However, it is apparent that there must be limits to the reduction of extension of the cavity. Rotary cutting instruments have remained the technique of choice for cavity preparation since GV Black but in recent years there have been other techniques suggested,[44] such as air abrasion and lasers. There remains some doubt as to whether these are necessarily more accurate and efficient and research must continue. Whilst patient comfort is of importance, preservation of natural tooth structure is paramount.

## CONCLUSIONS

It is suggested that it is now appropriate that the paradigm as proposed by GV Black be consigned to history and the profession should accept and apply the knowledge currently offered by science on the subject of cariology and treatment of the caries lesion. As Black has already pointed out “the complete divorcement of dental practice from studies of the pathology of dental caries, which has existed in the past, is an anomaly in science that should not continue”.[4] The profession must now move on and accept a new paradigm which begins with the understanding that the disease is the dominant factor. Elimination of the disease is of primary significance and restoration of the damage caused will then become secondary and can be carried out in a far more conservative manner than it has been in the past. It is accepted that changes of such dimension will be a challenge to the profession and will take time to implement. The real question is whether the profession is prepared to take the lead and make the change because it will involve considerable modification to the way the discipline of operative dentistry is currently practiced. The changes will need to be adopted by all the parallel professions related to dentistry as well as governments and those parties in the insurance industry that undertake fee support for our patients. Diagnosis of each patient should begin with an investigation into the oral environment in general, beginning with recognition of the microbial flora, saliva flow and control of the intra-oral pH levels. The patient needs to be convinced of the presence of the disease and to understand that they are the only ones able to control it. Techniques for the detection of the disease and level of pathology are now available but require further research to improve the level of sophistication so that the disease can be visually demonstrated to the patient. The whole concept of minimal intervention dentistry becomes paramount wherein the optimum result of control of the disease results from minimal intervention from the dentist. As progress of a caries lesion is generally quite slow there is normally time during which the emphasis must be on disease control.[45] Minimal cavity designs combined with sealing of doubtful fissures should be undertaken over this period because it is apparent that the bacterial flora cannot be controlled in the presence of open lesions. Where there is a high level of caries activity there may be a need to place transitional restorations using bioactive materials that will lead to remineralization of deeper lesions and help stabilize the bacterial flora.[46] The period of time required for effective monitoring of disease activity has yet to be clearly identified but should be spread over at least one year in the presence of high activity. Much will depend upon the operators ability to educate the patient but there is no doubt failure to eliminate the disease will lead to continuation of the problem.

Where there is a need for extensive rehabilitation the disease condition must be stabilized first before an overall treatment plan can be formalized and restorations placed with an expectation that they will be permanent in the true sense of the word. After all, replacement of any restoration will involve further loss of natural tooth structure and this is a finite commodity. The essential requirements of a permanent restoration include restoration of the true anatomy of the tooth crown to allow freedom in the envelope of occlusal movements[47] along with proper protection of the surrounding periodontal tissues. The current emphasis on aesthetics should remain secondary to maintaining a stable wear factor over the expected life of the patient. At the present time gold opposed to gold will offer the best level of stability. Ceramic restorations in general should only be opposed by ceramic and the resin composites and glass-ionomers need to be improved. The significance of in vivo observation and research cannot be overemphasized and the profession has a serious responsibility to insist on improved longevity with all restorative materials. As suggested by Black, operative dentistry must begin with cariology and it is essential that it become again the dominant discipline in this profession. With elimination of the disease and the consequent reduction in the damage caused by the disease there will be a commensurate reduction in the surrounding disciplines and our patients will be healthier and happier. The entire practising profession will need to be involved in the introduction of these changes in philosophy. This must begin with the new students as they enter the profession while the older practitioner will require retraining through continuing education courses that emphasize the basic premise of this paradigm, i.e., that it is essential to begin treatment of the patient with elimination of the disease prior to considering repair of the damage caused by the disease.
